# Supplementary material for: Thiol-Functional Polymer Nanoparticles via Aerosol Photopolymerization
Source: Polymers (Basel). 2021 Dec 13;13(24):4363. doi: 10.3390/polym13244363 (PMC8704326; doi:10.3390/polym13244363)
Supplement: Supplementary file 1 [file polymers-13-04363-s001.zip › polymers-1496695-supplementary.pdf]

polymers

Article

Thiol-Functional Polymer Nanoparticles via Aerosol Photopolymerization

Narmin Suvarli, Iris Perner-Nochta, Jürgen Hubbuch, and Michael Wörner *

Karlsruhe Institute of Technology (KIT), Institute of Process Engineering in Life Science, Section IV: Biomolecular Separation Engineering, 76131 Karlsruhe, Germany; narmin.suvarli@kit.edu(N.S.); iris.perner.nochta@kit.edu (I.P.-N.); juergen.hubbuch@kit.edu (J.H.)

* Correspondence: michael.woerner@kit.edu

Supporting information

I. Nuclear Magnetic Resonance.

Experimental. Solid state ^{13}C magic angle spinning (MAS) NMR was carried out with solid polymer nanoparticles in Bruker double channel 4 mm probe with a spinning frequency of 12 kHz using Bruker Avance 400 NMR spectrometer (B_0 = 9.4 T (Larmor frequency $\nu_0(^{13}C)$ = 100.63 MHz and $\nu_0(^{1}H)$ = 400.17 MHz). ^{13}C Single Pulse MAS NMR experiments were performed with a $\pi/4$ pulse duration of 2.9 microseconds and a 60 seconds recycling delay; these recording conditions ensure the quantitative determination of the proportions of the different carbon species. Typically, 1800 scans were recorded. ^{13}C CPMAS NMR experiments were acquired using a ramp for Hartmann-Hahn matching with a 5 seconds recycling delay and a contact time of 2 milliseconds. The radiofrequency field strength used for ^{1}H decoupling was set to 69 kHz. Chemical shifts reported relative to tetramethylsilane (TMS).

Results. The solid state ^{13}C NMR spectra of TMPTA homopolymer and Trithiol-TMPTA heteropolymer and Trithiol-TATT heteropolymer nanoparticles (**Figure S1**) show that thorough cross-linking of TMPTA took place in the Trithiol-TMPTA heteropolymer. TMPTA homopolymer still shows a peak of carbons of unreacted double bonds at 129.51 ppm. The peak of carbons located near the free -SH groups was not identified due to the broad peak and overlap at 20-42 ppm range. The polymer from Trithiol and TATT produces polymer with unreacted double bonds at δ 149.92 ppm. The following data corresponds to the ^{13}C NMR spectra in **Figure S1**.

Product of aerosol photopolymerization of TMPTA: Solid state 13C NMR (400 MHz), δ 7.65, 18.87, 24.36, 41.64, 66.37, 129.51, 165.87, 174.42.

Product of aerosol photopolymerization of Trithiol and TMPTA: Solid state 13C NMR (400 MHz), δ 8.24, 20.52, 27.15, 34.48, 41.58, 64.56, 171.81.

Product of aerosol photopolymerization of Trithiol and TATT: Solid state 13C NMR (400 MHz), δ 8.28, 28.92, 35.60, 42.67, 65.63, 149.92, 172.01.

II. Fourier Transform Infrared Spectroscopy

Presence of -SH groups in Trithiol, M1, P1 and P5 were measured using a Fourier transform infrared spectrometry utilizing attenuated total reflectance (FTIR-ATR, Equinox 55, Bruker Optics), and the spectra are presented in **Figure S2**. The spectra of M1 and P1 are combined with offset to show the presence of -SH groups on P1 and absence in M1. The spectrum of Trithiol is combined with the spectrum of P5 to show absence of -SH groups in P5. In general, detection of -SH groups is not completely reliable in this case, due to very weak absorption of -SH stretching mode even in the sample of Trithiol.

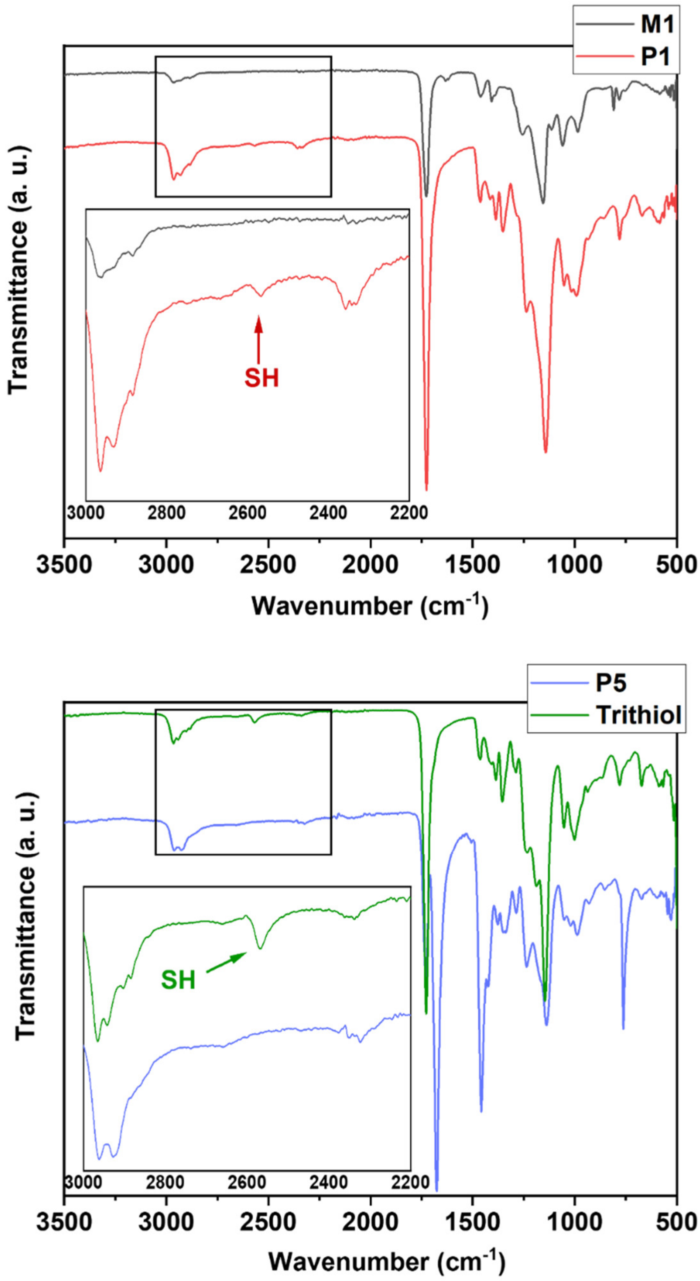

Figure S1. Solid State 13C NMR spectra of a) Trithiol-TMPTA heteropolymer, b) TMPTA homopolymer, c) Trithiol-TATT heteropolymer nanoparticles. Possible structures of the polymers are presented on the right side of the corresponding spectrum.

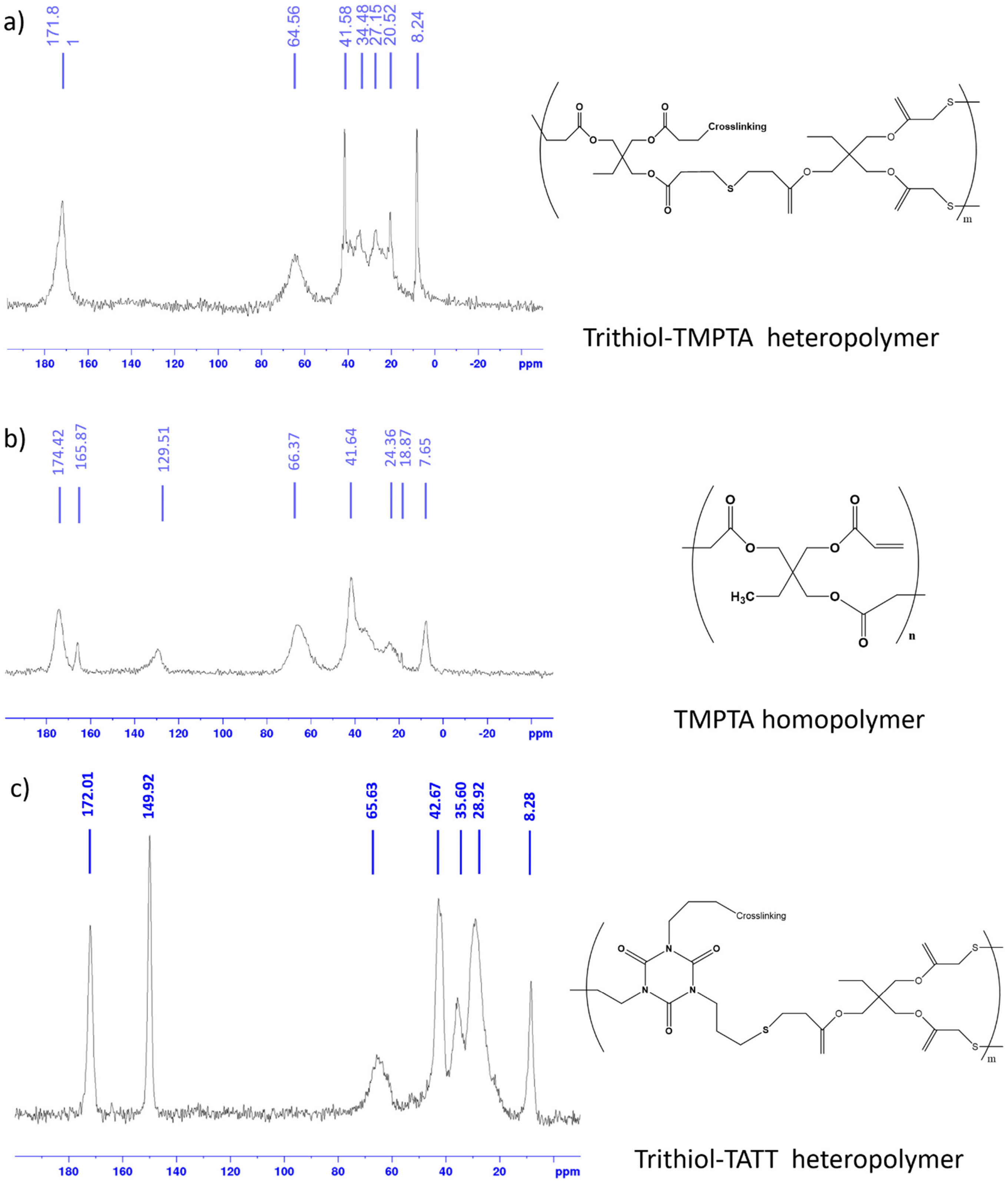

Figure S2. FTIR spectra of the TMPTA homopolymer (M1), Trithiol-TMPTA heteropolymer (P1), Trithiol-TATT heteropolymer (P5) nanoparticles and Trithiol monomer. The spectra are offset to show the presence of -SH groups in Trithiol and P1 nanoparticles and their absence in M1 and P5.

III. Aerosol photopolymerization of acrylates and thiol-ene monomers.

The formulations of other spray solutions of thiol-ene monomer combinations are presented in **Table SI.** The parameters are chosen to be comparable with experiments described in the main part: solvent ratio is 1:1 to the ratio of combined monomers, the quantity of the photoinitiator corresponding to 1wt% of combined monomers. TMPTA and NPG are the only alkene monomers among the tested alkenes that produce polymer nanoparticles via aerosol photopolymerization (M1, M2). The homopolymer nanoparticles of TMPTA and NPG are presented in **Figure S3**.

Table SI. Formulations of spray solutions used in aerosol photopolymerization, presenting monomers, their quantities and the employed solvent.

Spray solution	Thiol	Thiol (mM)	Alkene	Alkene (mM)	Solvent
M1	-	-	TMPTA	33.7	EtOH
M2	-	-	NPG	47.1	EtOH
P16	TMPIC	12.1	TMPTA	12.1	MeCN
P17	TMPIC	12.1	TEG-DVE	18.1	MeCN
P18	TMPIC	11.6	DAA	17.3	MeCN
P19	TMPIC	11.8	NPG	17.8	MeCN
P20	Trithiol	15.7	TMPTA	12.6	EtOH
P21	Trithiol	13.0	TMPTA	16.3	EtOH
P22	Dithiol	24.7	TATT	16.5	EtOH

Several thiol-ene monomers produced individual polymer nanoparticles in aerosol photopolymerization reaction, whereas some others produced aggregated materials (**Figure S4**). During this research it was supposed that AcO affected the properties of the nanoparticles, i.e., may cause increased agglomeration. In the main text of this paper, we described that some thiol-ene combinations were dissolved in AcO to ensure homogeneous spray formulations. To test, whether the change of solvent (from AcO to MeCN) affects the degree of agglomeration of polymer nanoparticles, combinations of TMPIC with alkene monomers were also combined with MeCN in spray solution formulations. The SEM images of produced nanoparticles (P16-P19) revealed no significant

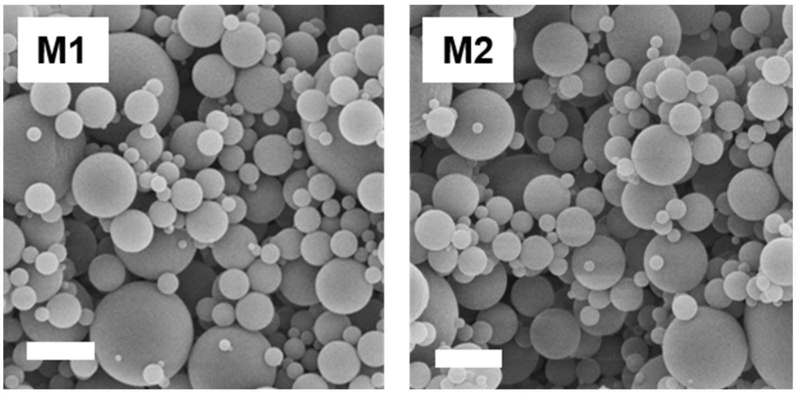

Figure S3. SEM images of homopolymer nanoparticles produced via APP of TMPTA (left) and NPG (right).

difference to SEM images of P5-P9. This evinces that AcO did not cause the agglomeration of nanoparticles in combinations P8 and P9. The nanoparticles from the combination of TMPIC and TMPTA are individual in every used solvent (P6 and P16).

Aerosol photopolymerization of thiol-ene monomers with unequal stoichiometric ratios of functional groups was carried out as well (P20 and P21). Ratios of TMPTA and Trithiol were shifted in favor of Trithiol (P20) and TMPTA (P21). Polymer nanoparticles in P20 start to form agglomerates when the ratio of functional groups changes from 1:1 to 5:4 (with 25% increase in favor of Trithiol in sample P20). When the ratio of thiol is increased to 50%, a premature polymerization reaction takes place inside the aerosol generator and no polymer nanoparticles could be collected on the filter membranes. An increase in thiol amount in the aerosol photopolymerization reaction with TMPTA leads to agglomeration; the reason for this behavior can be the presence of more unreacted thiol groups in the polymer nanoparticles after the APP process triggering post polymerization reactions.

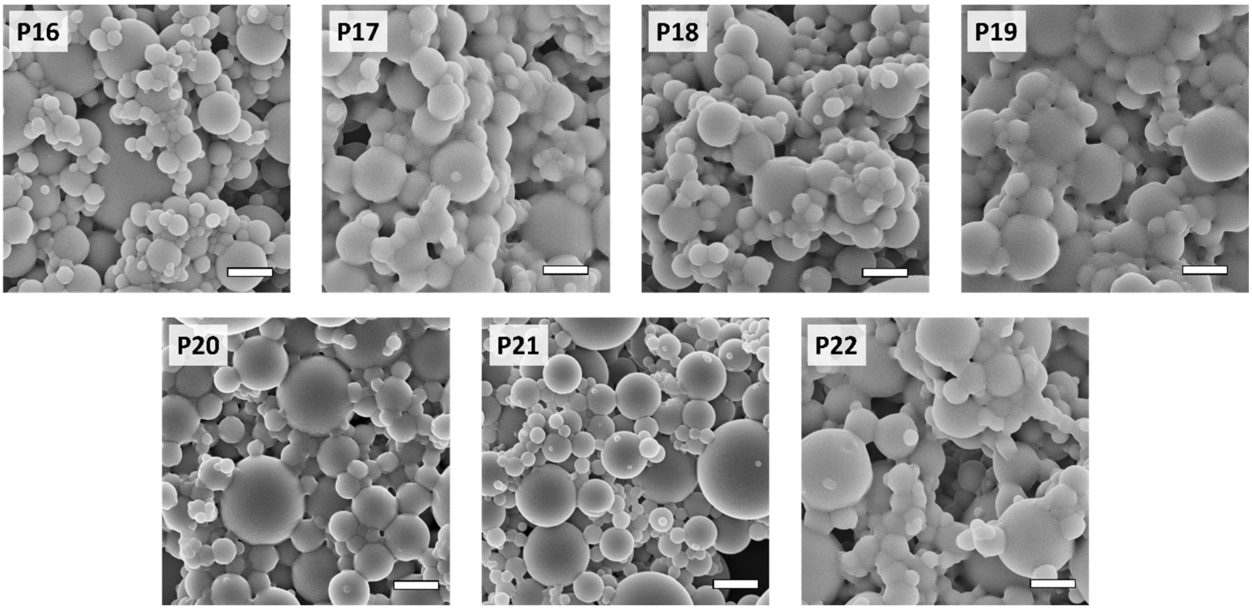

Figure S4. SEM images of polymer nanoparticles produced via aerosol thiol-ene photopolymerization. The nomenclature on the top left of each micrograph corresponds to spray solution formulations in Table I. Scalebar - 1μm.

When the balance of ratio of monomers is shifted towards TMPTA in the APP, polymer nanoparticles appear individual (P21).

Dithiol did not form polymer nanoparticles with TEG-DVE and DAA, however a premature polymerization reaction takes place in combinations of Dithiol with TMPTA and NPG starting inside the aerosol generator. Polymer nanoparticles cannot be produced from combination of Dithiol and DAA and TEG-DVE via APP, although an oily material is collected. In fact, the thiol-ene photopolymerization reaction may occur within a few seconds [1, 2]. Nevertheless, the reactivity of alkenes in the thiol-ene photopolymerization depends on the chemical structure of the alkene and has to be considered [3].

The combination of Dithiol and TATT (P22) produced a small amount of collected product before the premature polymerization occurred in the aerosol generator after 45 minutes of atomization. Dithiol and TATT combination resulted in formation of polymer nanoparticles which show significant agglomeration (P22). We expect that cross-linking takes place between the nanoparticles on the filter membrane after the aerosol photopolymerization reaction is finished or during sample preparation for SEM analysis.

References:

1. Jasinski, F., et al., *Thiol–Ene Linear Step-Growth Photopolymerization in Miniemulsion: Fast Rates, Redox-Responsive Particles, and Semicrystalline Films.* Macromolecules, 2016. **49**(4): p. 1143-1153.
2. Jasinski, F., et al., *Light-Mediated Thiol–Ene Polymerization in Miniemulsion: A Fast Route to Semicrystalline Polysulfide Nanoparticles.* ACS Macro Letters, 2014. **3**(9): p. 958-962.
3. Northrop, B.H. and R.N. Coffey, *Thiol–Ene Click Chemistry: Computational and Kinetic Analysis of the Influence of Alkene Functionality.* Journal of the American Chemical Society, 2012. **134**(33): p. 13804-13817.
